# Value of brain injury-related indicators based on neural network in the diagnosis of neonatal hypoxic-ischemic encephalopathy

**DOI:** 10.1515/biol-2022-0686

**Published:** 2023-08-30

**Authors:** Lijun Wang

**Affiliations:** Zhengzhou Institute of Industrial Application Technology, Zhengzhou 451100, Henan Province, China

**Keywords:** neural network, brain injury-related indicators, diagnosis of neonatal hypoxic ischemic encephalopathy, clinical medicine, Apgar score, cord blood gas analysis

## Abstract

Neonatal hypoxic ischemic encephalopathy is a common disease, which is caused by fetal hypoxia, asphyxia, and other reasons. It may cause sequelae of the nervous system and even death in children. Computer tomography examination can clarify the scope and location of the disease and provide the basis for clinical treatment and prognosis. Relevant personnel analyzed the symptoms of ischemic hypoxia and found that ischemia and hypoxia were the main causes of encephalopathy. Neonatal ischemia and hypoxia are easy to cause serious damage. At present, with the development of medicine, the function of the human brain is the most important issue in natural science. The law of neural activity and the role of molecular cells, organs, and systems have fundamental construction significance for the prevention and treatment of nerve and mental diseases. By analyzing the value of the diagnosis of neonatal hypoxic-ischemic encephalopathy in the analysis of experimental data, by setting the newborns in the controlled group and the trial group as experimental subjects, this paper analyzed the situation of newborns in terms of body temperature, body weight, and respiratory rate, and used Apgar score to score these standards. It was found that the score of the controlled group was 7 and above, and the score of the trial group was below 7. It was found that the Apgar scoring method was more simple. Then, the newborns were analyzed by cord blood gas analysis. It was found that most of the data in the control group were between 7.8 and 8.4, and the data in the trial group were between 5.8 and 7.1. The umbilical blood gas analysis score of the experimental group was lower than that of the control group. By comparing the satisfaction of cord blood gas analysis and the Apgar score, it was found that the satisfaction of cord blood gas analysis was 24.06% higher than that of the Apgar score.

## 1 Introduction

Hypoxic-ischemic brain damage is a common neonatal disease. Due to the incomplete development of children’s body tissues, brain tissue damage caused by hypoxia and other reasons is very serious. Hypoxic ischemic brain damage is an important factor in the onset of neonatal disease. Relevant studies have shown that the location of the disease has a great relationship with age, and the results and treatment principles vary with age. Early detection and preventive measures play an important role in reducing neonatal mortality. Neonatal hypoxic-ischemic encephalopathy is caused by ischemic hypoxia, and the main reason is hypoxia, which is easy to cause fetal life risk. In the process of production, abnormal fetal heart rate and placenta are easy to cause hypertension in pregnant women and prenatal fever, and thus lead to difficulties in production.

In the past, the diagnosis was mainly based on the history of hypoxia, clinical symptoms, and imaging, and imaging examination was the necessary means of diagnosis. Mollayeva Tatyana has studied and focused on neurological issues, including fragility and injury treatment, and gender trends. In the context of neural problems, two different methods were analyzed [1]. Edlow et al. put forward new ideas on advanced horizontal neuroimaging technology, identified the brain network of patients, and provided directions for diagnosis and prediction. They also activated the damaged neural network and radiated new potential, discussed the latest progress in detection and prediction, and provided opportunities to improve the symptoms of patients [2]. Hutchinson et al. detected brain tissue, improved quantitative methods, and assessed the pathological mechanisms, and caused neurobiological complexity research. The physical environment information was provided for the study of microstructure characteristics, the implementation of the constructed model, and the interpretation of measurements [3]. Cronberg et al. assessed the patient’s nervous system, combined with the analysis of neurologists and physiologists, and found that excessive prediction would lead to serious disease status. If the patient was not treated in time, it would lead to the patient missing the timely treatment opportunity. At present, the prediction tools include physiological tests and chemical image biomarkers. It is necessary to clear sedative drugs after drug withdrawal and provide sufficient information for patients to recover [4]. The neural network has good advantages in the diagnosis of central nervous system diseases and can comprehensively reflect pathological changes. It is an important research field of medical research.

In the application of neonatal ischemic hypoxic encephalopathy, the diagnosis, and treatment of neonatal ischemic hypoxic encephalopathy were analyzed. Wang et al. surveyed medical management by using the method of horizontal survey and used the methods of statistics and chi-square analysis to test. They analyzed the cost and case proportion of different hospitals and adopted appropriate methods to make plans for the growth of infants, providing opportunities for the long-term neurological development of infants [5]. Lundgren et al., to determine the risk factors and incidence rate of infant hypoxic-ischemic encephalopathy, found that infant hypoxic-ischemic encephalopathy (HIE) was related to acute emergency and delivery mode through a large number of experimental data and investigations. Risk assessment can reduce the occurrence of risks and reduce medical errors [6]. Novak et al. found that hypoxic-ischemic encephalopathy was related to the time point. By determining the risk factors of unknown causes and comparing the incidence rate and related cases, data analysis was carried out on the incidence rate of newborns and placental tissues. In multivariate regression, the confidence interval and function were analyzed [7]. Ouwehand et al. predicted the neural outcome of infants with hypoxic-ischemic encephalopathy and the diagnostic accuracy of different models for the treatment of neural development results. By using the literature retrieval technology and through the analysis of experimental data, the influencing factors of neuro dysplasia and nerve death were predicted for neural development [8]. During the duration of hypoxia and asphyxia, the self-regulation ability of brain tissue would be affected, resulting in poor blood circulation of the brain. This would lead to brain tissue ischemia and hypoxic injury.

The above studies have only carried out a separate study on the brain injury-related indicators of neural networks and the diagnosis of neonatal hypoxic-ischemic encephalopathy, without combining the two. Although these studies have some references, they are more or less insufficient to demonstrate the conclusion and have some room for improvement. To solve the value of brain injury-related indicators of neural networks in the diagnosis of neonatal hypoxic-ischemic encephalopathy, this paper adopted the method of experimental analysis, compared the data of body temperature, body weight, and respiratory rate, and analyzed the trial group and the controlled group. The experimental setup in this paper was simple to operate.

## 2 Evaluation of neonatal HIE

### 2.1 Development status of neonatal HIE

Ultrasound is the most common clinical diagnostic method at present, but its sensitivity and resolution are very poor. It is difficult to find systemic lesions, and the surgical effect is also affected by the subjective factors of the operator. The low density of white matter in the brain cannot be used as a diagnostic standard in intracranial Computer Tomography (CT) examination. In the left hemisphere and other parts of the brain, intracerebral hemorrhage appears as small patches or dots of high signal. Because multisequence imaging is rich in image information, it can clarify the nature of the disease and help improve the accuracy of diagnosis. The diagnosis has high resolution and can display gray matter signals, and can more accurately display intracranial anatomical structure, which is helpful for clinical diagnosis and treatment, and provides a theoretical basis for the treatment of central neuralgia after spinal cord injury.

Although image scanning is a common examination method, which can display brain edema, cerebral hemorrhage, and other diseases, it has a certain reference value for the diagnosis of brain edema and cerebral hemorrhage. However, the brain tissue of newborns is not fully developed, which would have a great impact on the radiation of brain cells. By analyzing the relationship between brain concussion, brain tissue injury, and neurodegeneration, relevant scholars found that the risk of chronic traumatic encephalopathy increased. However, because the current research results have no relevant reference value, they proposed the consequences of repeated neurobehavior and the prediction of neurodegeneration pathology. When evaluating animals, balance function and cognitive behavior are measured. It lays a foundation for neurology and protein research, which proves that the axis mutation of cortical proteins and the presentation of neuritis are relatively low [9].

Hypoxic-ischemic encephalopathy causes damage to the nervous system of infants. The main causes of distress are the problems of the uterus and the abnormal function of the uterus. Relevant experts have sorted out independent data sets and mapped the location of nerve injury into a map to test the relationship between depression and lesion location. The validation evaluation data set is used to improve the efficiency of the test, and no brain region is affected. In the brain circuit, the prefrontal cortex is the center, which is excluded from the verification. Later, it was found that the depression circuit came from the brain injury and stimulation sites, which promoted the improvement of later depression [10]. Exploring the clinical influencing factors of hypoxic-ischemic encephalopathy provides a theoretical basis for clinical treatment, prevention, and nursing.

### 2.2 Factors affecting neonatal HIE

Because of the disorder of brain material metabolism and the damage of nerve cells, it has good diagnostic value for clinical treatment. In addition, the examination can also find small lesions, such as small lesions in the white matter area, simple white matter edema, and high sensitivity. In particular, the detection rate of brain diseases has been greatly improved, and diagnostic accuracy has been greatly improved. Relevant scholars selected several healthy infants as the research object. Because with the growth of fetal age, the gray matter, white matter, and brain tissue of newborns have a trend of increasing. There is no significant difference between mild and moderate values of neonatal hypoxic-ischemic encephalopathy. The application of this method in neonatal hypoxic-ischemic encephalopathy can early detect the condition of children.

The development of neonatal encephalopathy is not yet fully mature, and it is very sensitive to hypoxia and other factors. Neonatal hypoxia, asphyxia, and other factors are likely to lead to neonatal hypoxia, and neonatal intrauterine distress is the main cause of neonatal disease. Relevant experts conduct quantitative analysis of data through a proteomic method to identify potential markers of neonatal hypoxic-ischemic encephalopathy. Bioinformatic analysis was conducted in the control group and the trial group to evaluate the characteristics and ability to identify the expressed protein. By analyzing the significantly upregulated levels of differentially expressed proteins and binding proteins, the recognition process of proteins is further studied, and potential biomarkers are obtained, reflecting the severity of the disease and playing a key role in the development of newborns [11]. There are many reasons for neonatal diseases, so the screening of newborns is particularly important. With the progress of medical technology, compared with the past, the mortality of newborns has been significantly reduced, but the incidence rate of brain injury, cerebral palsy, epilepsy, and other complications caused by apnea has increased by 5 percentage points. People should study the factors affecting ischemia and hypoxia to prevent and treat the disease and improve the curative effect.

Relevant experts discuss the incidence rate and mortality of severe hypoxic-ischemic encephalopathy. It is found that the proportion of patients is decreasing; the number of infant cases is gradually decreasing; the mortality rate is also decreasing, with no change in the severity. Since the obstetric characteristics remain unchanged and the resuscitation time is less, it is concluded that there is no correlation between the time and the change in obstetric characteristics [12,13]. In the application of neonatal ischemic hypoxic encephalopathy, clinical analysis of neonatal ischemic hypoxic encephalopathy is carried out. After the examination operation, the subjects observed the test results between the groups and compared the indexes of the examination between the groups. Through comparison, it is found that the indexes of the middle cerebral artery in the two groups are significantly higher. The examination is of great significance for the diagnosis of neonatal ischemic hypoxic encephalopathy and the clinical diagnosis and treatment of children.

**Figure 1:**
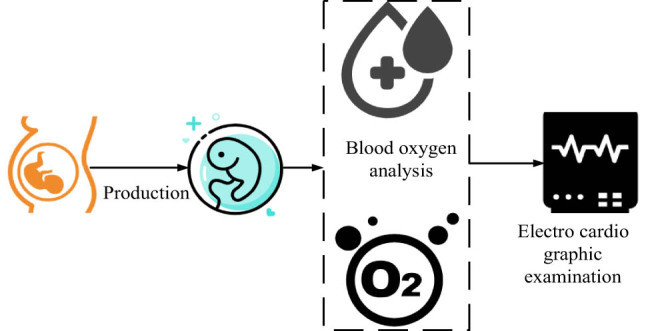
Influential factors of neonatal hypoxic-ischemic encephalopathy.

Relevant personnel analyze the relationship between vitamin D concentration and neonatal hypoxic-ischemic encephalopathy and evaluate the effect of vitamin D on surrounding tissues. The correlation coefficient is used to evaluate the correlation between clinical results, and standardized scoring is performed. After injecting high-dose vitamin D dose into infants, the wearing of respirators were analyzed. Data supports the relationship between low vitamin levels and adverse outcomes, and it is concluded that there is no relationship between vitamin D dose and neonatal hypoxic-ischemic brain injury [14]. The abnormality of the umbilical cord can cause fetal blood circulation disorder or interruption, resulting in fetal hypoxia or death. The development of various organs of the fetus is not perfect, and the mother’s temperature rises, which increases its basal metabolic rate, increases oxygen consumption, and significantly reduces the concentration of blood oxygen supplied to the fetus, resulting in fetal distress in the uterus [15]. In the early stage of the newborn, the heartbeat increases to ensure the normal blood supply of all organs and tissues, which leads to an increase in blood pressure. In the late stage of the disease, the level of middle cerebral artery disease increases significantly and the health factors decrease significantly. The influencing factors of neonatal hypoxic-ischemic encephalopathy are shown in Figure 1.

### 2.3 Value of brain injury-related indicators of neural network in diagnosis

Due to fetal distress caused by abnormal fetal heart rate monitoring, neonatal asphyxia can be caused, which is the direct cause of brain disease. Therefore, people should strengthen the prenatal examination and solve the fetal distress as soon as possible. The reason is that pregnant women could lead to fetal intrauterine hypoxia and insufficient cerebral blood supply. During pregnancy, pregnant women should regularly monitor their blood pressure to prevent pregnancy hypertension and eclampsia. Both conditions, such as placental abruption and placental aging, can cause insufficient oxygen supply to the fetus from the mother, resulting in ischemia and hypoxia. Thus, oxygen is transported from the pore space to the umbilical vein, resulting in fetal intrauterine hypoxia.

Relevant personnel analyze the behavior of human brain injury and induced brain injury. With the development of neural networks, the diseases of brain injury are reviewed; the advantages and limitations of injury are discussed; the clinical ecological relevance of the study is improved. Nerve injury is essential for the evaluation of neuroscience theory [16]. If the temperature of pregnant women continues to rise, the fetal heart rate would accelerate, which would lead to neonatal asphyxia. Therefore, patients with infectious fever in late pregnancy should end the pregnancy as soon as possible. Premature rupture of membranes (PROM) is a high-risk factor for infection in early pregnancy, but statistically, it has no significant impact. The main reason is that the time of PROM is short, and there is no serious infection. It would not lead to hypoxia. The severity of amniotic fluid pollution is related to the severity of hypoxia and ischemia.

Diabetes is the cause of blood vessel rupture and blood flow reduction, which is easy to cause fetal ischemia and hypoxia and affects the material exchange between mother and fetus. Due to the limited number of studies, the research results of blood vessels need to be further explored. The prenatal examination of pregnant women according to the time and statistical analysis of the age of the mother needs to be further tested. The factors affecting the incidence of ischemia and hypoxia are not significant due to the lack of choice between the trial group and the control group. Thus, oxygen is transported from the pore space to the umbilical vein, resulting in fetal intrauterine hypoxia.

**Figure 2:**
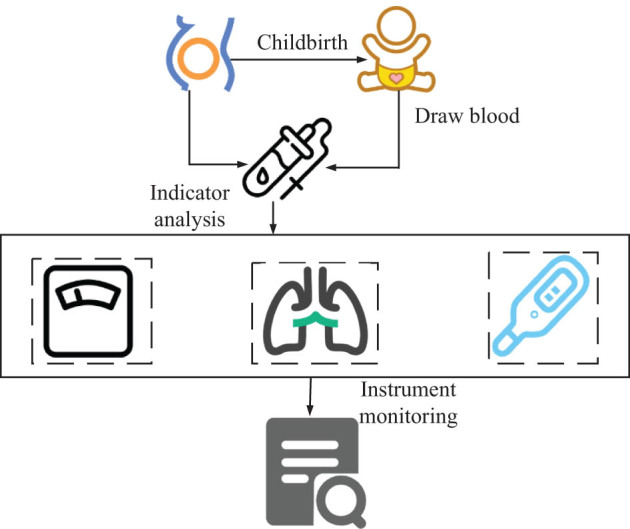
Analysis of the value of brain injury-related indicators of neural networks in the diagnosis.

Experts have studied the surgical drug resistance of epileptic patients and the damage model of hippocampal sclerosis. Due to the loss of neuron cells and the proliferation of glial cells, the brain would be temporarily damaged after the lack of seizure activity, and the excessive excitable neural structure and transmission mechanism would lead to the occurrence of epilepsy. Different from the traditional experimental study of epilepsy, to further understand the pathogenesis, including different types of injury, human hippocampal biopsy is used to conduct a detailed analysis of animal models, transform new methods, and open up prospects for global patients [17]. Through regression analysis, it is found that the factors affecting neonatal ischemia and hypoxia are very different, and a comprehensive analysis is needed to determine some factors affecting neonatal disease. The value analysis of brain injury-related indicators of neural networks in the diagnosis is shown in Figure 2.

The high-risk factors during pregnancy are mainly maternal diseases, such as placenta, placental abruption, PROM, and umbilical cord encirclement. These diseases are prone to complications during pregnancy. At the same time, medical workers should understand its pathogenesis and risk factors, strengthen professional theory and basic operating skills, do a good job in perinatal education, strictly monitor the production process, and correctly handle various complications. Relevant personnel proposes a fully automatic organization segmentation method to establish a variable model and improve segmentation efficiency. A segmentation pipeline is established, which uses high-resolution tissue classification technology to optimize the 3D modeling output and maintain the musculoskeletal surface. The fully automatic segmentation method provides good segmentation technology. The segmentation accuracy needs to be improved with the most advanced methods to display the functional segmentation performance in different tissue morphology and quantitative images [18]. Brain edema, softening, necrosis, atrophy, and intracranial hemorrhage are the manifestations of brain tissue metabolic disorders, cell damage, and brain blood insufficiency caused by perinatal hypoxia.

Symptoms of hypoxia and ischemia are prone to produce different results. Prevention of mild patients can effectively avoid severe cases. Severe patients may cause irreversible damage to brain tissue, which may lead to the death of children. Serious cases may even lead to the early death of newborns. The content of glycogen in the newborn’s brain is low, and nutrition is mainly absorbed through blood circulation and umbilical cord transmission. Insufficient nutrition can easily affect the development of the newborn’s nervous system and limbs. After the newborn is born, it is necessary to carry out a physical examination on the newborn, which is prone to perinatal ventricular rest symptoms. In the early stage of the disease, the increase of blood pressure would lead to the damage of the self-regulation mechanism of the brain blood, resulting in the low blood circulation speed of the brain, and hypoxia and ischemia symptoms. Neonatal intracerebral hemorrhage occurs more frequently in the left and right hemispheres, and the stimulation of the cerebral cortex has problems, resulting in necrosis and ulceration of brain tissue, and echo phenomenon in brain tissue.

Since children are prone to blood vessel rupture and brain edema after being hit, the complex phenomenon of meridians may be related to the mechanism obstacle of hypoxia and ischemia. Children’s brain symptoms are mainly manifested by brain fog, local flakes and dots, and narrowing and blurring of brain gullies. In the late stage of the disease, the brain is prone to brain atrophy and brain structural changes, which can easily lead to sequelae. The reason for these symptoms is that the central nervous system is not fully developed in anatomical biology, resulting in diffuse brain phenomenon. Therefore, in the study of hemodynamics, it is necessary to consider the blood blockage of cerebral arteries and veins, which would lead to a decrease in the number of glial cells and the gray myelination of nerve cells, resulting in cerebral hemorrhage and cerebral infarction. Cerebral ischemia and hypoxia are the causes of serious diseases, so in daily life, people should pay attention to normal living habits and work and rest to ensure the normal operation of the brain.

## 3 Experimental evaluation of brain injury-related indicators

### 3.1 Experimental method

About 200 newborns were divided into two groups. Among them, 100 were newborns with hypoxic-ischemic encephalopathy; the other 100 were normal newborns; the sick newborns were the trial group; the healthy children were the controlled group. The weight, temperature, and pulses of 200 newborns were recorded, and the experimental results were compared and analyzed.


**Informed consent:** Informed consent has been obtained from all individuals and included in this study.
**Ethical approval:** The research related to human use has been complied with all the relevant national regulations and institutional policies and in accordance with the tenets of the Helsinki Declaration and has been approved by the authors’ institutional review board or equivalent committee.

### 3.2 Observation indicators

Five pairs of trial groups and the controlled group were set up to analyze the weight, body temperature, and pulse of newborns with Apgar score, observe and record the data of the trial group and controlled group, and compare them.

Trial group: Apgar score was used to analyze the observation indexes of neonates with hypoxic-ischemic encephalopathy. After instrument observation and recording, the experimental data were analyzed and compared.

Controlled group: Apgar score was used to analyze the observation indexes of normal newborns, and the data range was recorded and observed.

### 3.3 Statistical processing

All statistical data of the trial group and the controlled group were input into the Statistical Product and Service Solutions statistical software, which expressed the measured value with mean ± standard deviation (±*S*). Assuming that the test level is *a* = 0.05, *P* < 0.05 indicates a statistical difference.

## 4 Evaluation of experimental results of related indicators of hypoxic-ischemic brain injury

### 4.1 Control evaluation of newborn weight

Five pairs of experimental data were selected to compare the trial group and the controlled group, and the experimental results were analyzed. The control analysis of newborn weight is shown in Table 1.

**Table 1:** Comparative analysis of newborn weight

Category	Group	Data				
		1	2	3	4	5
Weight	Control group (kg)	3.2 ± 0.2	3.5 ± 0.1	3.7 ± 0.2	4 ± 0.3	2.8 ± 0.1
	Trial group (kg)	2.2 ± 0.2	2.3 ± 0.3	2.1 ± 0.2	4.4 ± 0.2	4.2 ± 0.2
	Control group Apgar score	8	7	9	8	7
	Trial group Apgar score	5	6	4	5	4

The newborn weight range of the controlled group was 2.7–4.3 kg; the Apgar score was 7 or above; the newborn weight range of the trial group was 1.9–4.6 kg; the Apgar score was less than 7; the range of the trial group and the controlled group after using Apgar score was 4–9. Because the Apgar score was lower than 7 points, it proved that the newborn in the trial group was in distress. The weight of newborns in the trial group was in a dangerous range compared with those in the control group, so it can be concluded that the Apgar scoring method was more simple and convenient.

### 4.2 Comparison of neonatal respiratory rate

In the experiment, the Apgar score was used to record the respiratory rate per minute of newborns in the controlled group and the trial group, and the differences between the controlled group and the trial group were compared. The comparison of neonatal respiratory rate is shown in Table 2.

**Table 2:** Comparison of neonatal respiratory rate

Category	Group	Data				
		1	2	3	4	5
Respiratory rate	Control group	42 ± 3	44 ± 4	40 ± 5	45 ± 2	43 ± 3
	Trial group	50 ± 4	53 ± 2	55 ± 3	35 ± 5	37 ± 4
	Controlled group Apgar score	8	9	8	7	9
	Trial group Apgar score	5	6	5	6	6

By observing the experimental data, it can be found that the Apgar score was used to measure the respiratory rate of the trial group and the control group. Comparing the experimental results, in the controlled group, the respiratory rate of the newborn was in the range of 39–48 per min, and in the trial group, the respiratory rate of the newborn was in the range of 30–58. After analyzing the data, it was found that in the comparative analysis of neonatal respiratory rate records using the Apgar score, the respiratory rate of newborns with ischemia and hypoxia in the trial group was not within the normal range, and drug treatment was needed.

### 4.3 Evaluation of neonatal temperature

This paper observed the data of five pairs of trial groups and a controlled group and compared the temperature analysis before and after the experiment. The analysis and comparison of neonatal body temperature are shown in Table 3.

**Table 3:** Analysis and comparison of neonatal body temperature

Category	Group	Data				
		1	2	3	4	5
Thermoanalysis	Control group (°C)	36.7 ± 0.2	36.5 ± 0.3	36.4 ± 0.2	36.9 ± 0.4	36.7 ± 0.2
	Experimental group (°C)	37.2 ± 0.2	37.8 ± 0.2	38.4 ± 0.2	37.4 ± 0.2	38.5 ± 0.2
	Controlgroup Apgar score	8	9	8	7	8
	Experimental group Apgar score	4	6	5	6	3

Through observation, it was found that the temperature of newborns in the trial group was high and low, which was basically in the range of 37–38.7°C, while the temperature of newborns in the controlled group was in the normal range, which was basically in the range of 36.2–37.3°C. The Apgar score of newborns in the controlled group was 7–9 points, and the Apgar score of newborns in the trial group was 3–6 points. Because the Apgar score indicates that the risk of neonatal asphyxia is more serious, it is necessary to carry out real-time detection of newborns during production to avoid overheating or hypothermia.

### 4.4 Cord blood gas evaluation

**Figure 3:**
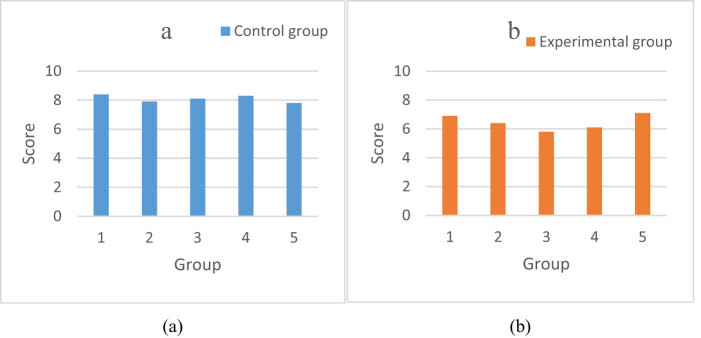
Analysis and comparison of cord blood gas: (a) controlled group and (b) trial group.

Five groups of data were selected to evaluate the effect of umbilical cord blood gas analysis on newborns in the trial group and the control group, and the data were collected and compared. The specific data are shown in Figure 3.

As can be seen from Figure 3, Figure 3a is the umbilical blood gas analysis score of the controlled group, and Figure 3b is the umbilical blood gas analysis score of the trial group. According to the general trend and average data characteristics of the above two figures, it can be seen that the umbilical blood gas analysis score of the trial group was lower. Specifically, most of the data in Figure 3a were distributed between 7.8 and 8.4, with the highest value of 8.4, the lowest value of 7.8, and the average value of 8.1. The data in Figure 3b were between 5.8 and 7.1, with the highest value of 7.1, the lowest value of 5.8, and the average value of 6.46. The umbilical blood gas analysis score of the trial group was lower than that of the control group.

### 4.5 Cord blood gas evaluation and Apgar score satisfaction evaluation

**Figure 4:**
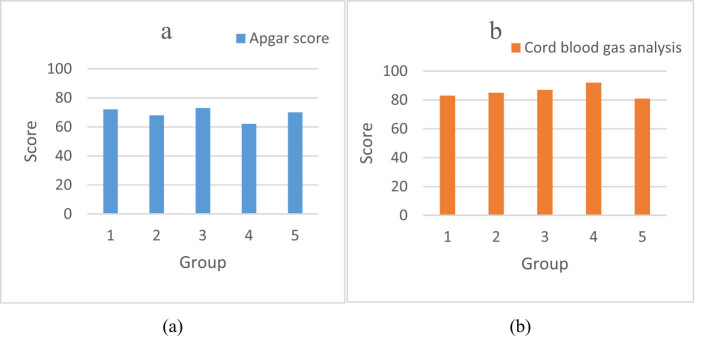
Cord blood gas analysis and Apgar score satisfaction analysis: (a) controlled group and (b) trial group.

By analyzing five groups of survey data, the satisfaction effects of cord blood gas analysis and Apgar score were compared. The cord blood gas analysis and Apgar score satisfaction analysis are shown in Figure 4.

From Figure 4, it can be seen that Figure 4a shows Apgar score satisfaction and Figure 4b shows cord blood gas analysis satisfaction. According to the general trend and average data characteristics of the above two figures, it can be seen that the analysis score of cord blood gas was higher. Specifically, most of the data in Figure 4a were distributed between 62 and 73, with the highest value of 73, the lowest value of 62, and the average value of 69. The data in Figure 4b was between 81 and 92, with the highest value of 92, the lowest value of 81, and the average value of 85.6. The satisfaction of cord blood gas analysis was 24.06% higher than that of the Apgar score.

To sum up, after comparing the effect satisfaction of cord blood gas analysis and Apgar score, it was found that cord blood gas analysis was more satisfactory and more convenient and accurate in solving neonatal hypoxic-ischemic encephalopathy. Because the umbilical blood gas analysis is the main evidence for analyzing the production and metabolism of newborns, and the main basis for evaluating the various indicators of the newborn body, the blood analysis, and acid-base condition of newborns can be used to analyze the severity of neonatal hypoxia, poisoning and asphyxia, and more intuitively explain the essential reasons. Compared with the Apgar score, cord blood gas analysis is more objective and accurate. After the birth of the fetus, the umbilical blood gas of the newborn is analyzed to understand the changes in various parameters. It is more helpful to find the asphyxia of newborns and reduce the death rate. By analyzing the postpartum situation of newborns, it is helpful to identify the occurrence of adverse symptoms and emergencies and avoid medical disputes. Umbilical blood gas analysis is listed as one of the essential indicators for the diagnosis of perinatal asphyxia, and it is of great significance to guide the treatment of newborns and judge the prognosis by organically combining with Apgar score and other indicators.

## 5 Discussions

Due to the implementation of the neonatal guardianship system, the incidence rate and critical rate of full-term infants have significantly decreased. After standardized treatment, a brain ultrasound examination was carried out. It was found that the echo of brain parenchyma recovered quickly, the volume of the brain decreased, and hydrocephalus and complications of the liquefied cystic cavity were less. Cerebral palsy, cognitive impairment, and abnormal hearing function are the main causes of white matter damage in premature infants. In a word, the reaction of the nervous system in children with brain damage is different due to the severity of brain parenchyma damage. Most mild children have no obvious sequelae, and moderate children can also be cured. A few children have hydrocephalus, encephalomalacia, encephalomalacia, cerebral atrophy, cerebral perforation, etc. Children have symptoms of mental retardation, epilepsy, cerebral palsy, etc. This method is simple, repeatable, and multiple bedside examinations. It is the best method for neonatal craniocerebral ultrasound examination and has a certain promotion value.

The condition of patients with craniocerebral injury changes rapidly, and some patients may even have more serious symptoms. It is necessary to monitor the changes in vital signs such as respiration, pulse, blood pressure, and body temperature of the patient at any time, and conduct CT and MRI examinations regularly. However, next to the hospital bed, there would also be a monitoring device to monitor the vital signs of patients. The most common is the intracranial pressure monitor, which places the probe on the surface of the brain or in the ventricle to monitor the intracranial pressure. If the intracranial pressure of the patient continues to rise, the prognosis of the patient would be poor, and active treatment must be carried out. People can also do cerebral blood flow monitoring. If the cerebral blood flow drops, it means that the patient has poor cerebral blood supply and cerebral ischemia. Brain metabolism monitoring can also be carried out, mainly including lactic acid, glucose, electrolytes, uric acid, and other indicators, and these indicators can reflect the metabolism of the brain. It can also monitor the cerebral oxygen partial pressure, observe whether there is anoxia in the brain, and monitor the cerebral artery oxygen partial pressure, thus indirectly reflecting the cerebral oxygen partial pressure.

It plays an important role in the process of apoptosis and plays a crucial role in the signal transduction pathway of apoptosis. It is the initial stage of apoptosis and one of the most important proteins that regulate and implement apoptosis. Due to ischemia and hypoxia, the human body needs a large amount of hemoglobin, so the demand for red blood cells would increase, which would promote the production and release of the hematopoietic system, leading to an increase in the content of peripheral blood. The method is simple and fast.

## 6 Conclusions

This paper studied the value of brain injury-related indicators of neural networks in the diagnosis of neonatal hypoxic-ischemic encephalopathy. By analyzing the development status of neonatal hypoxic-ischemic encephalopathy and the factors affecting neonatal hypoxic-ischemic encephalopathy, it showed the prospect of the diagnosis of neonatal hypoxic-ischemic encephalopathy. Through the analysis of the experimental data, it was found that the newborn weight in the controlled group ranged from 2.7 to 4.3 kg; the Apgar score was 7 or above; the newborn weight in the trial group ranged from 1.9 to 4.6 kg; Apgar score was below 7; the weight in the trial group was in the dangerous range. The Apgar scoring method was more simple and more convenient. Through the analysis of respiratory rate, it was found that the respiratory rate of the controlled group was in the range of 39–48. In the trial group, the respiratory rate of the newborn was in the range of 30–58. It was necessary to carry out respiratory detection for the hypoxic-ischemic newborn. In terms of body temperature, it was necessary to avoid the occurrence of asphyxia. To avoid the phenomenon of high or low temperature, through the comparison of umbilical blood gas analysis scores, it was found that the umbilical blood gas analysis score of the trial group was lower, which was lower than 7.1. By comparing the Apgar score and the satisfaction of cord blood gas analysis, it was found that the satisfaction of cord blood gas analysis was 24.06% higher than that of the Apgar score. This paper studied the value of brain injury-related indicators of neural networks in the diagnosis of neonatal hypoxia and ischemia. Through the experimental data, the value of various brain injury indicators in neonatal hypoxic-ischemic encephalopathy was analyzed. The content of this paper has reference significance for the prediction and prevention of neonatal diseases.
